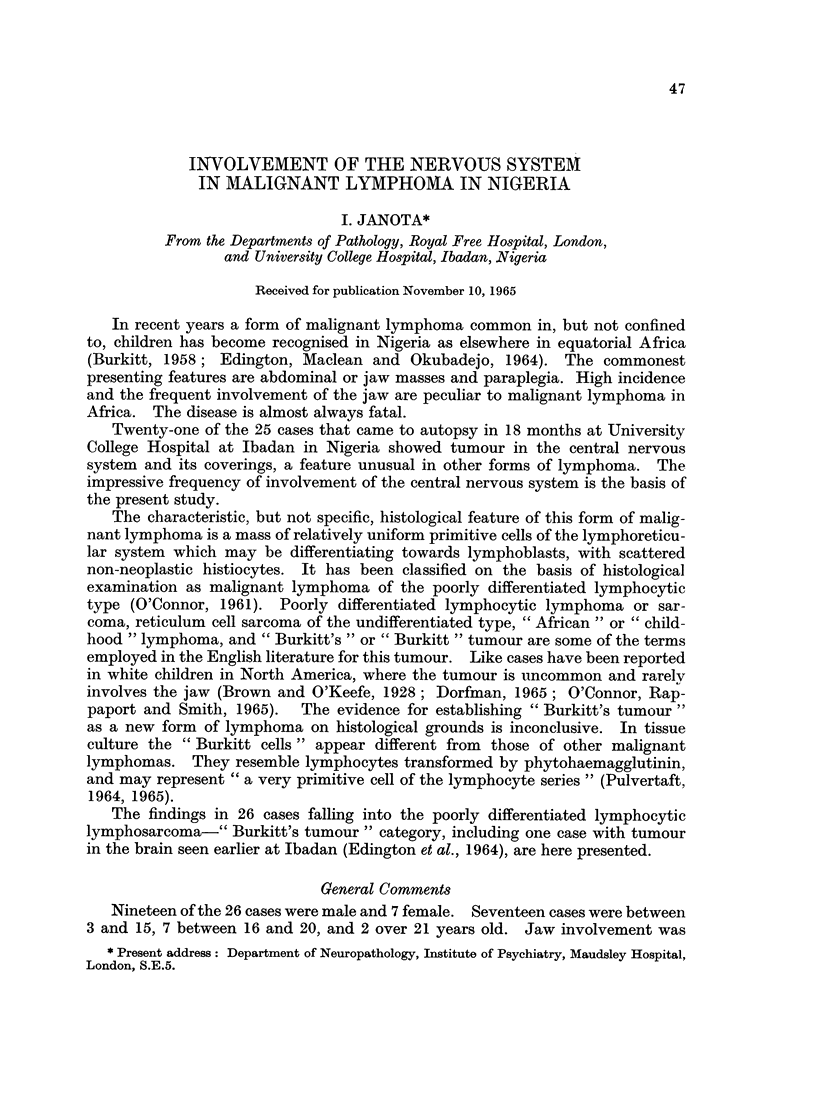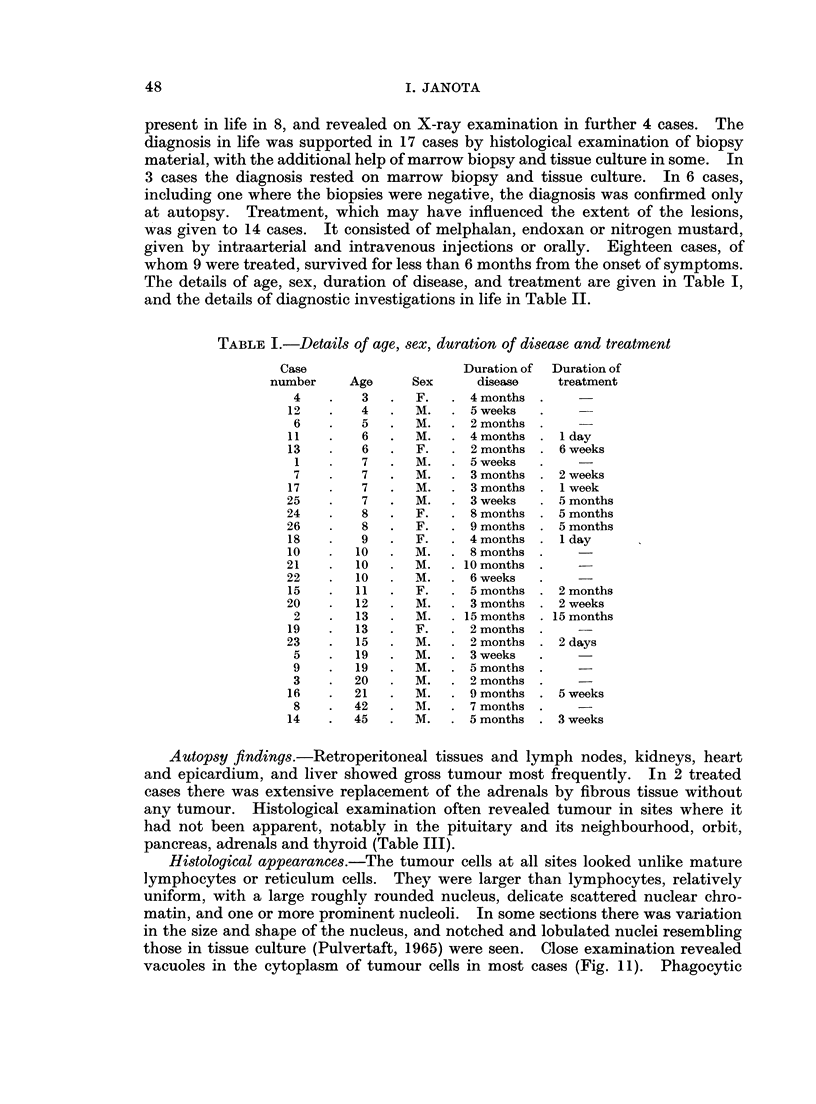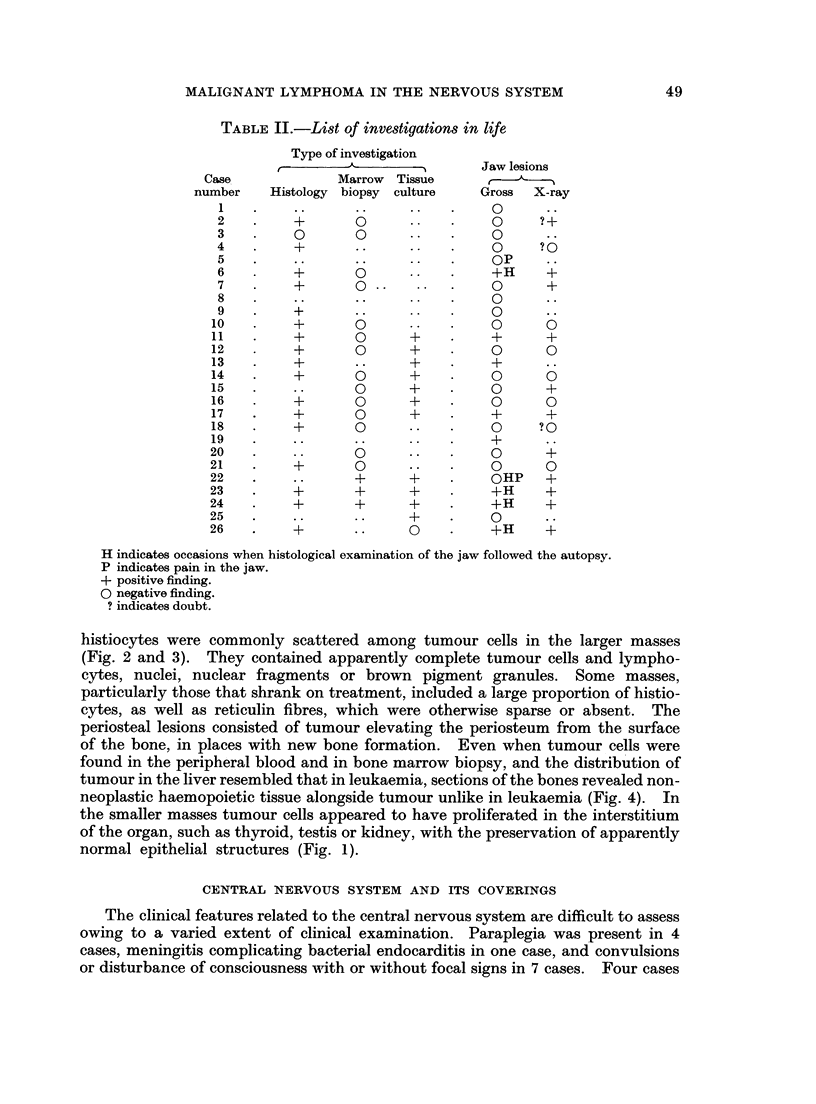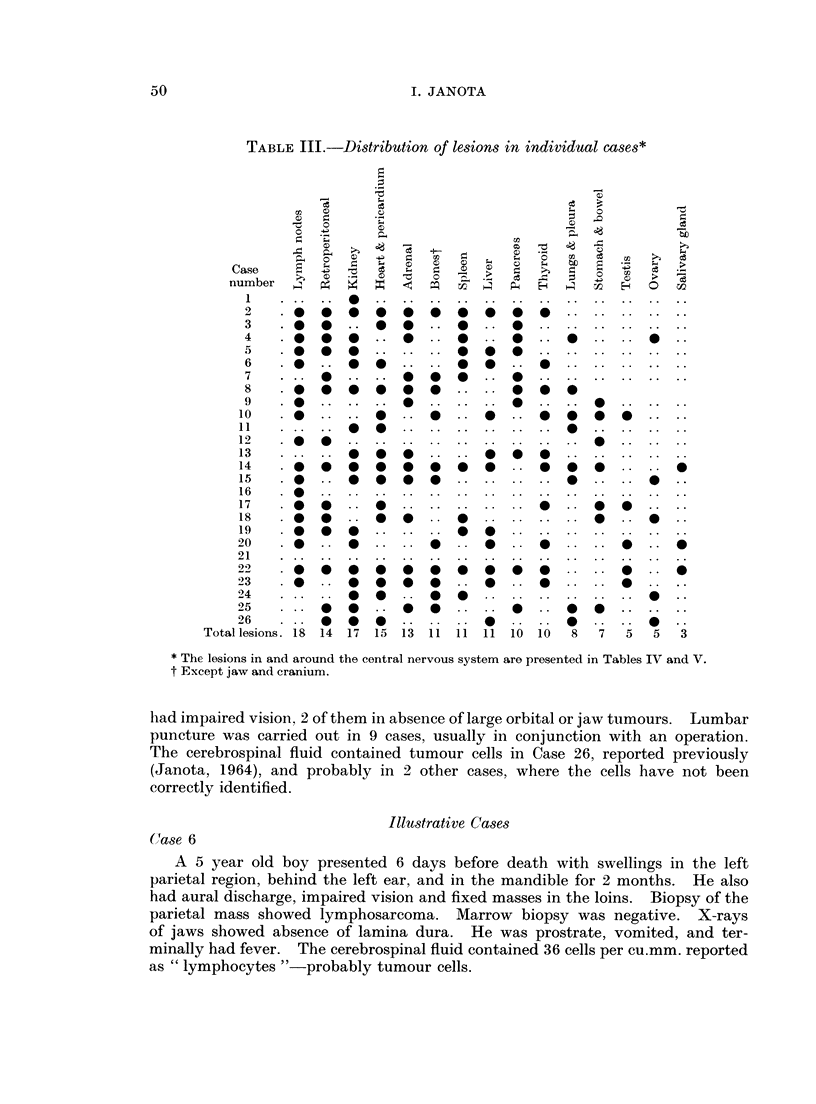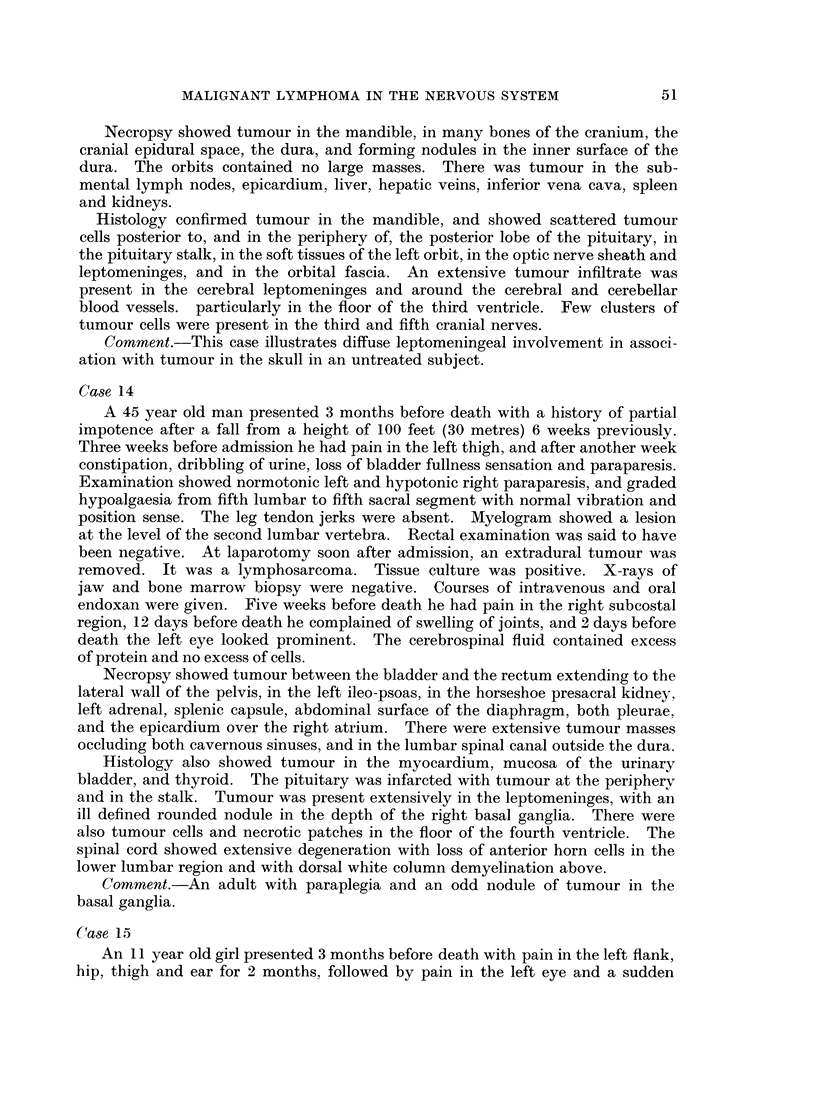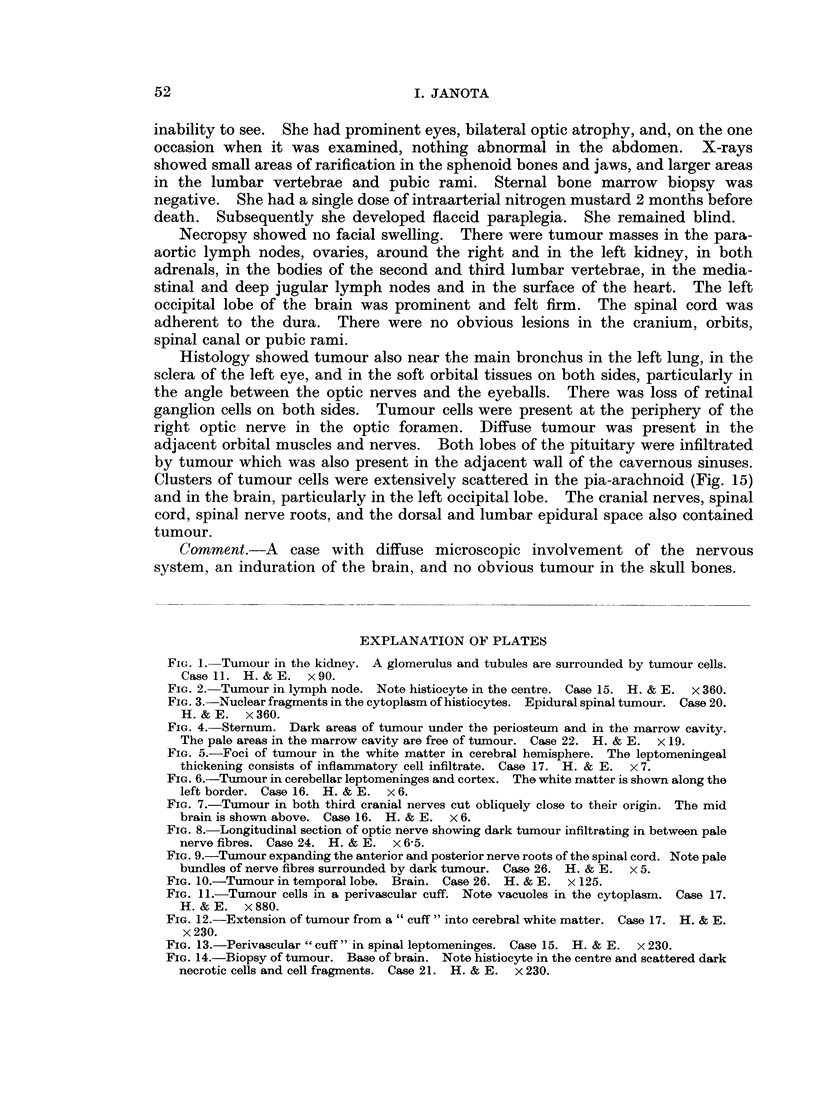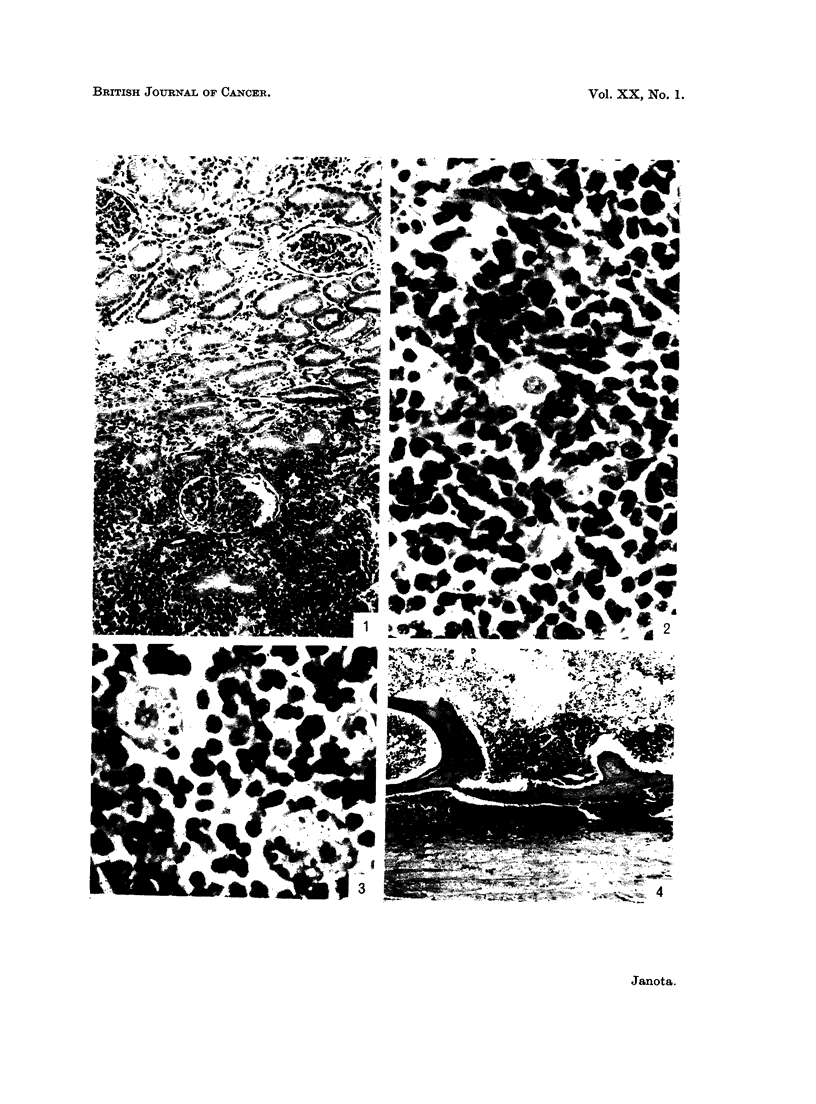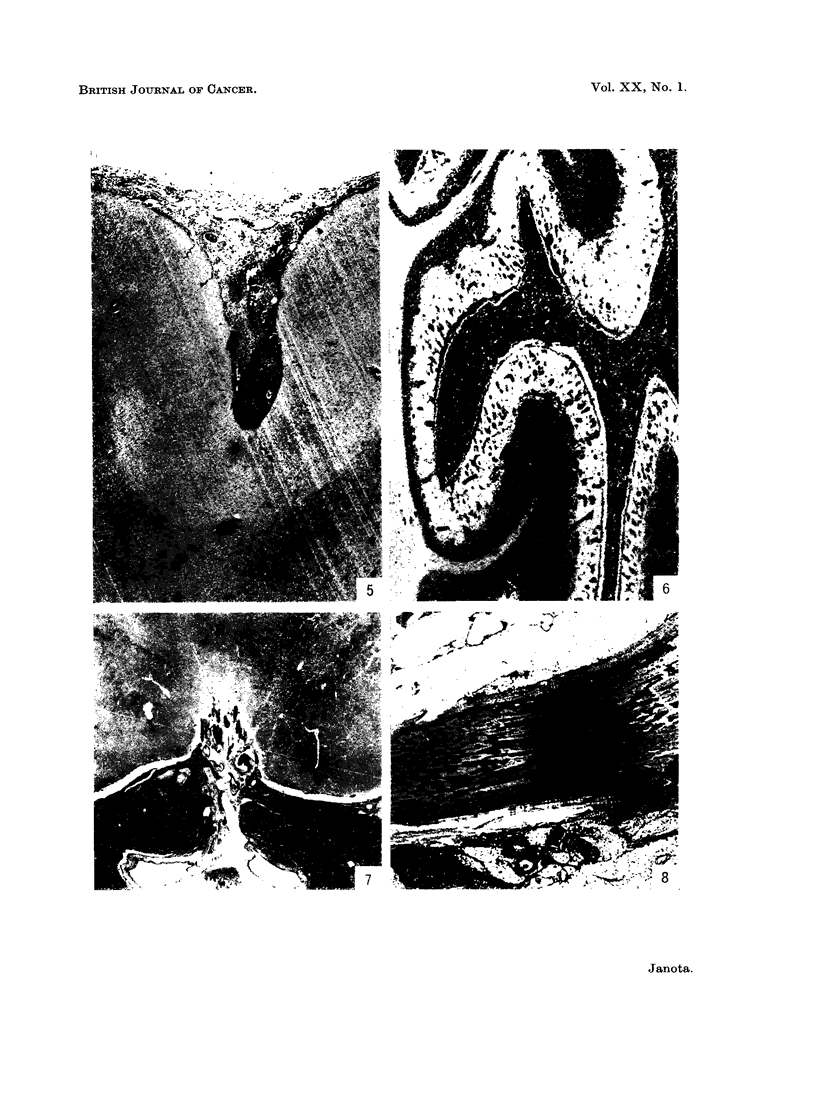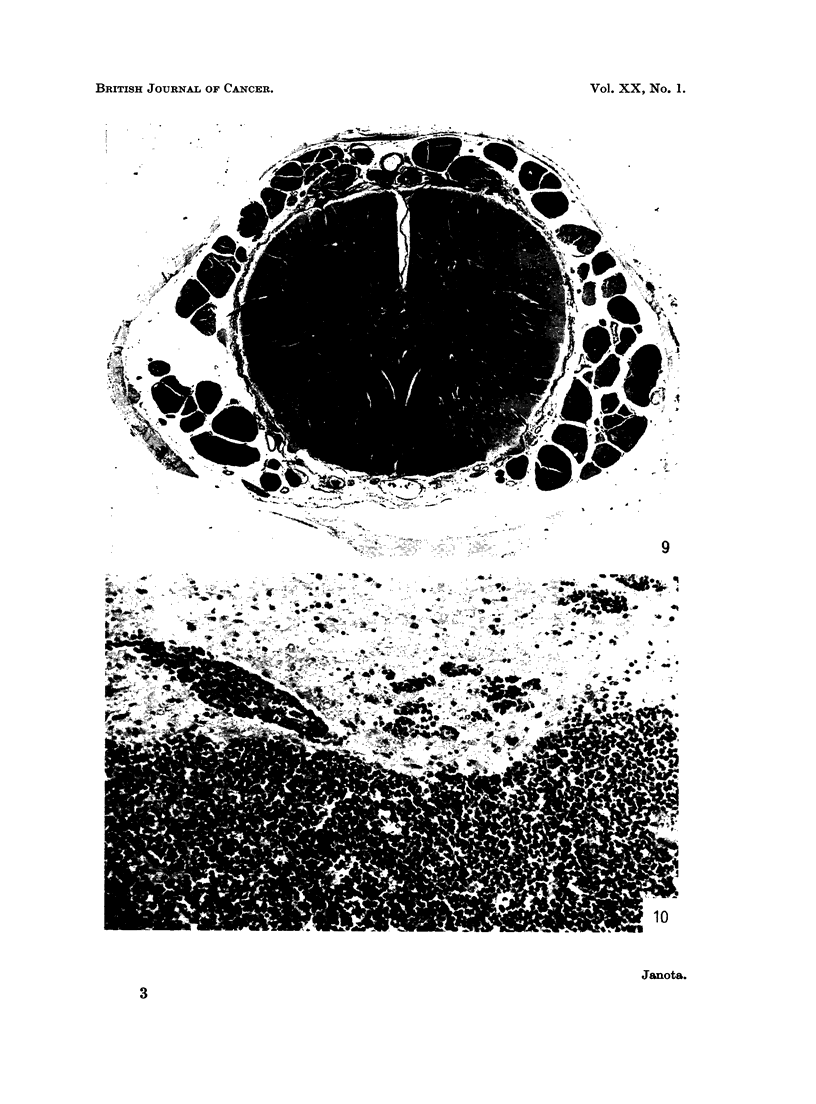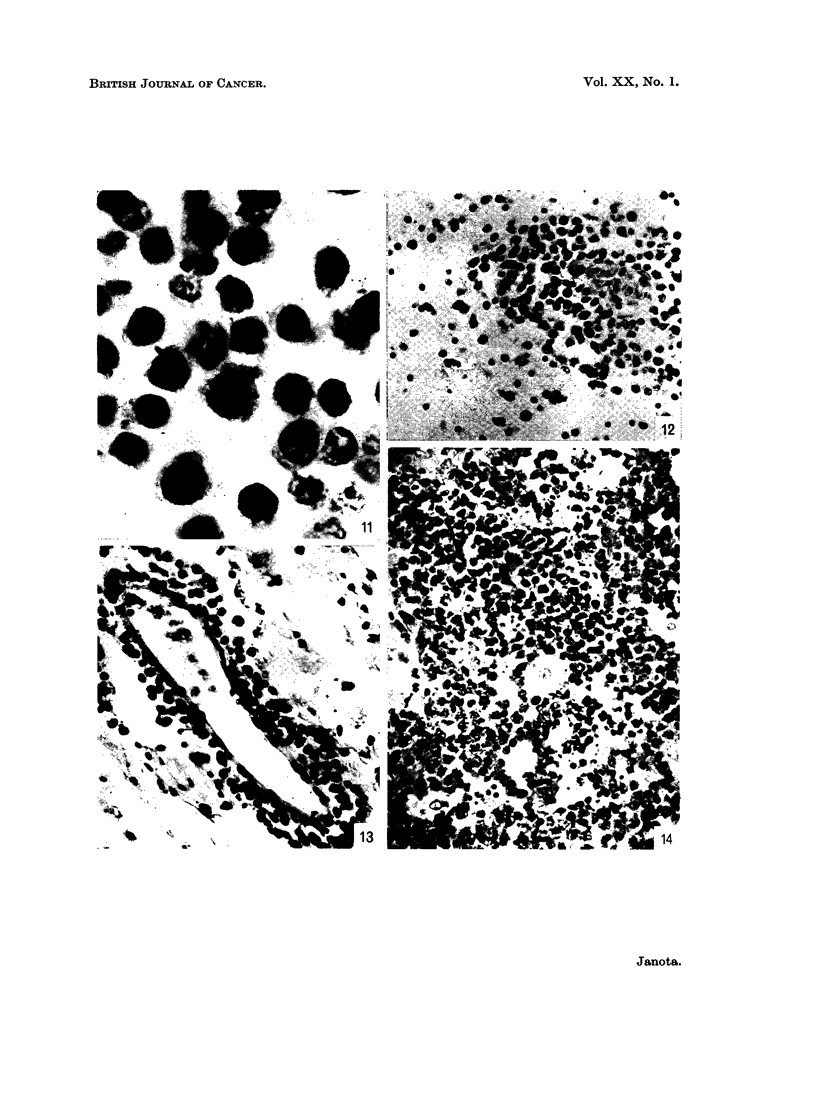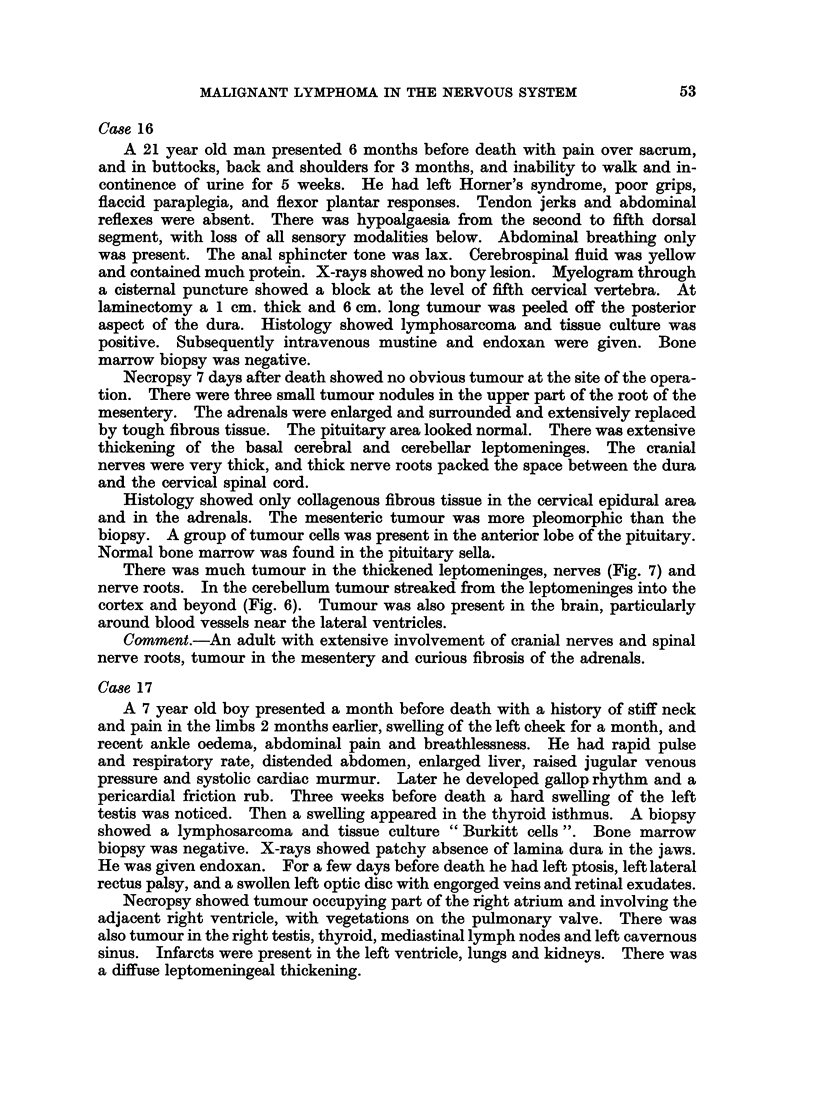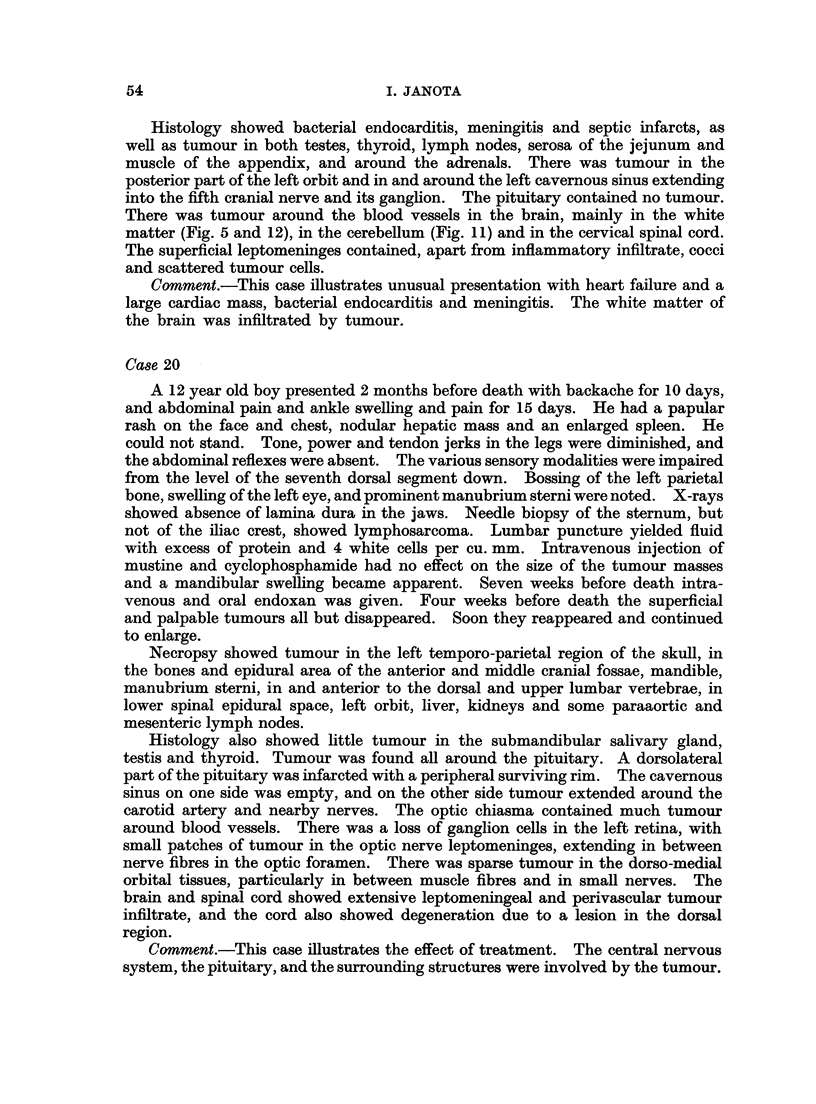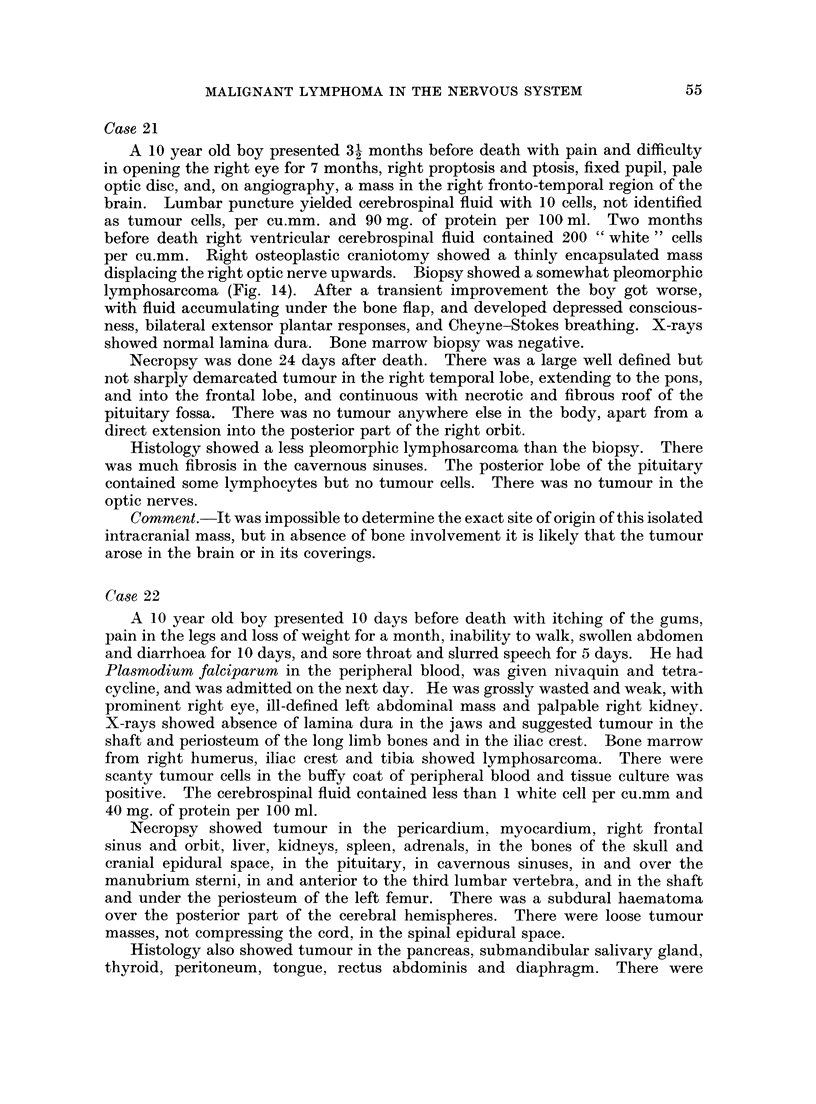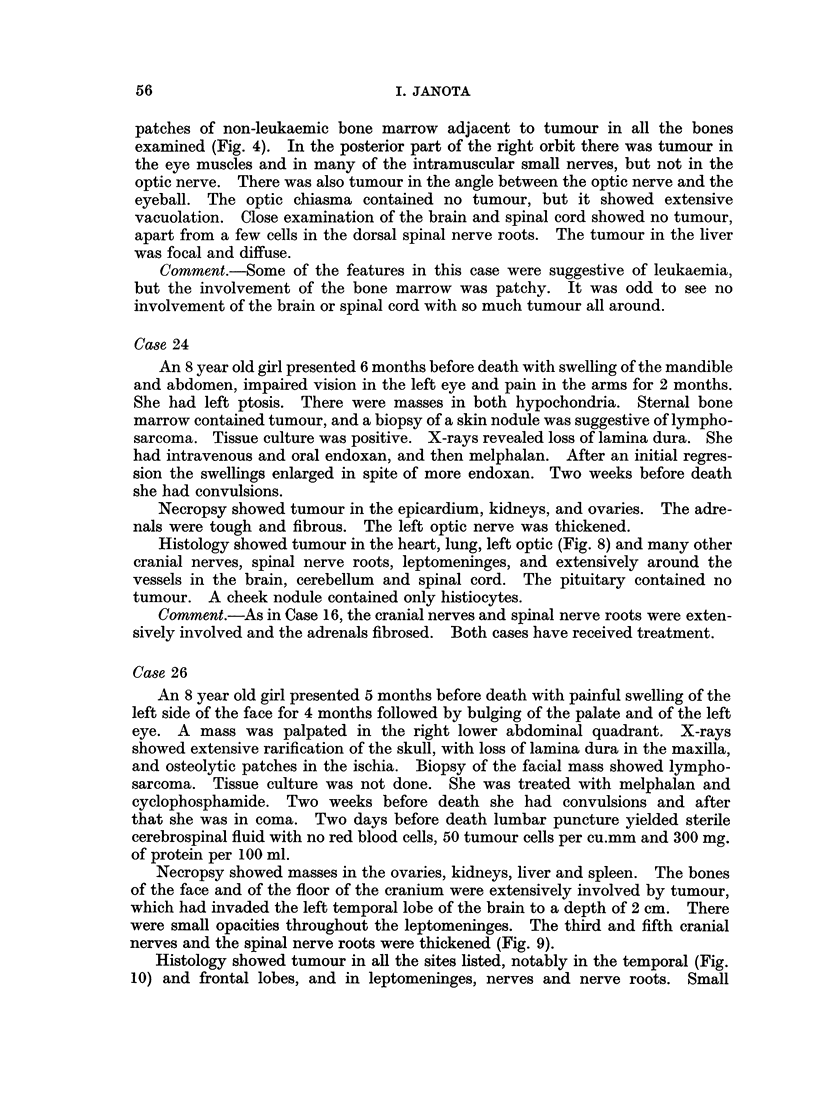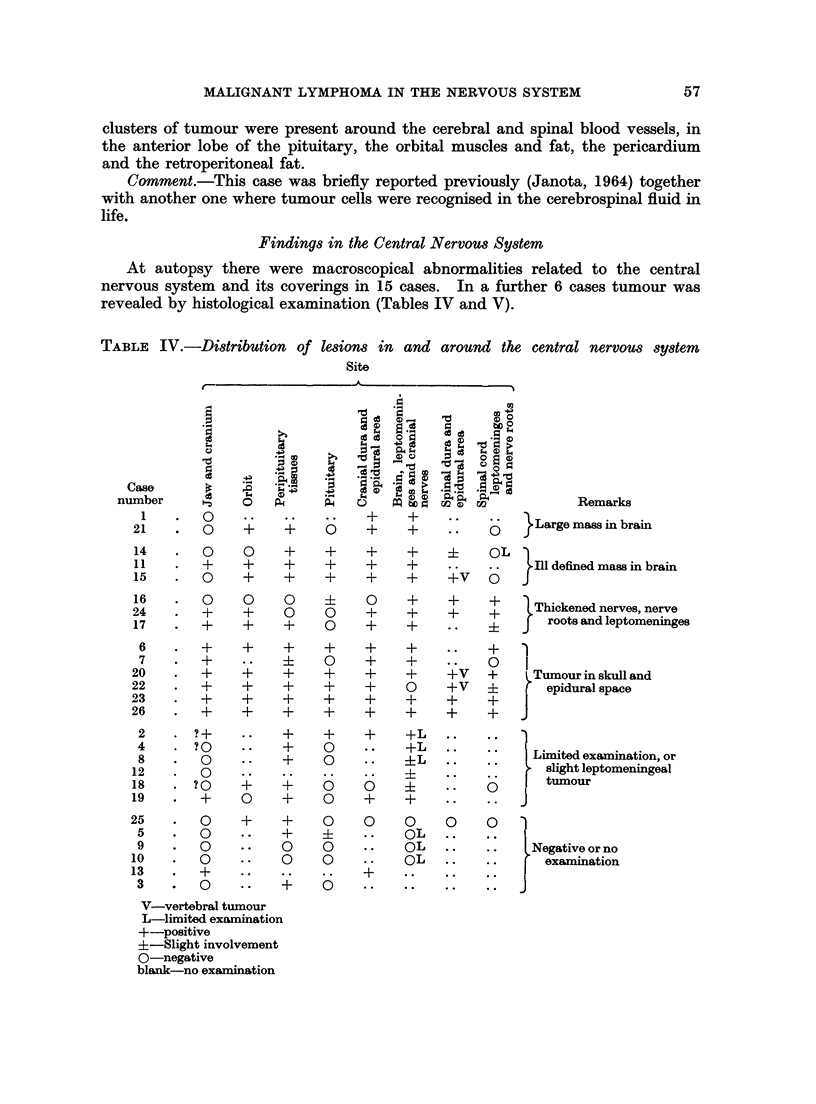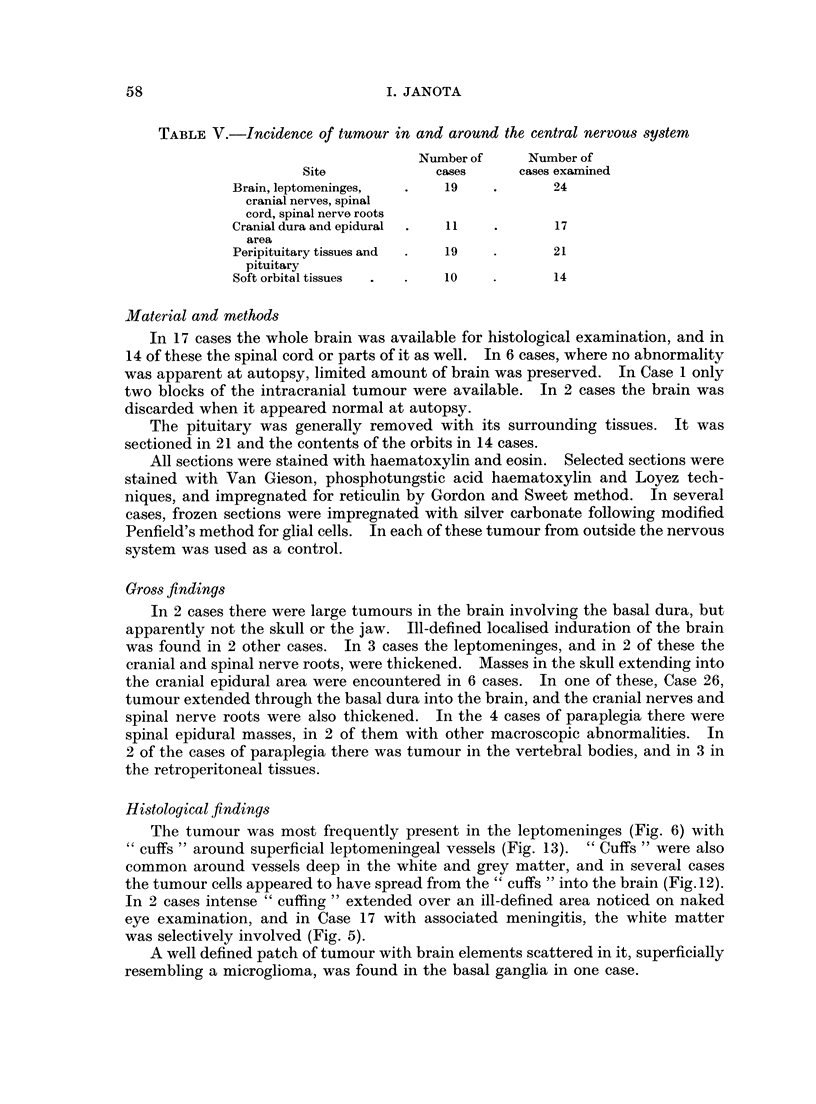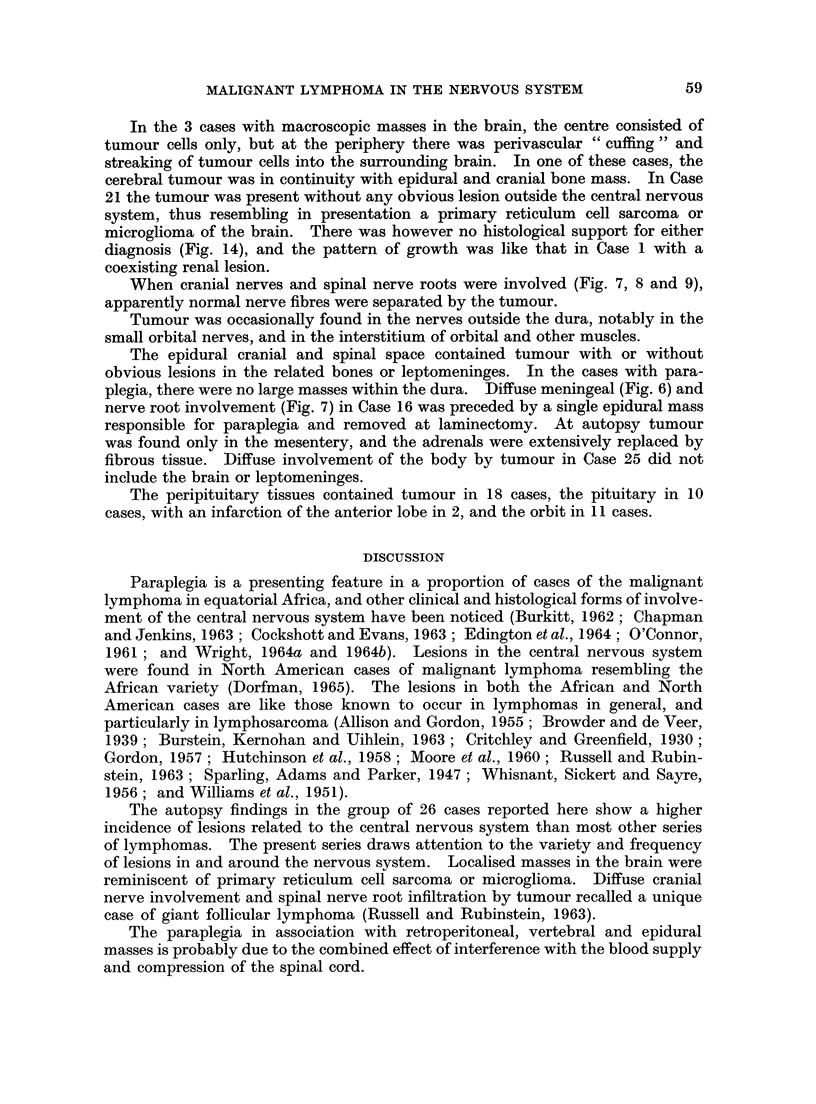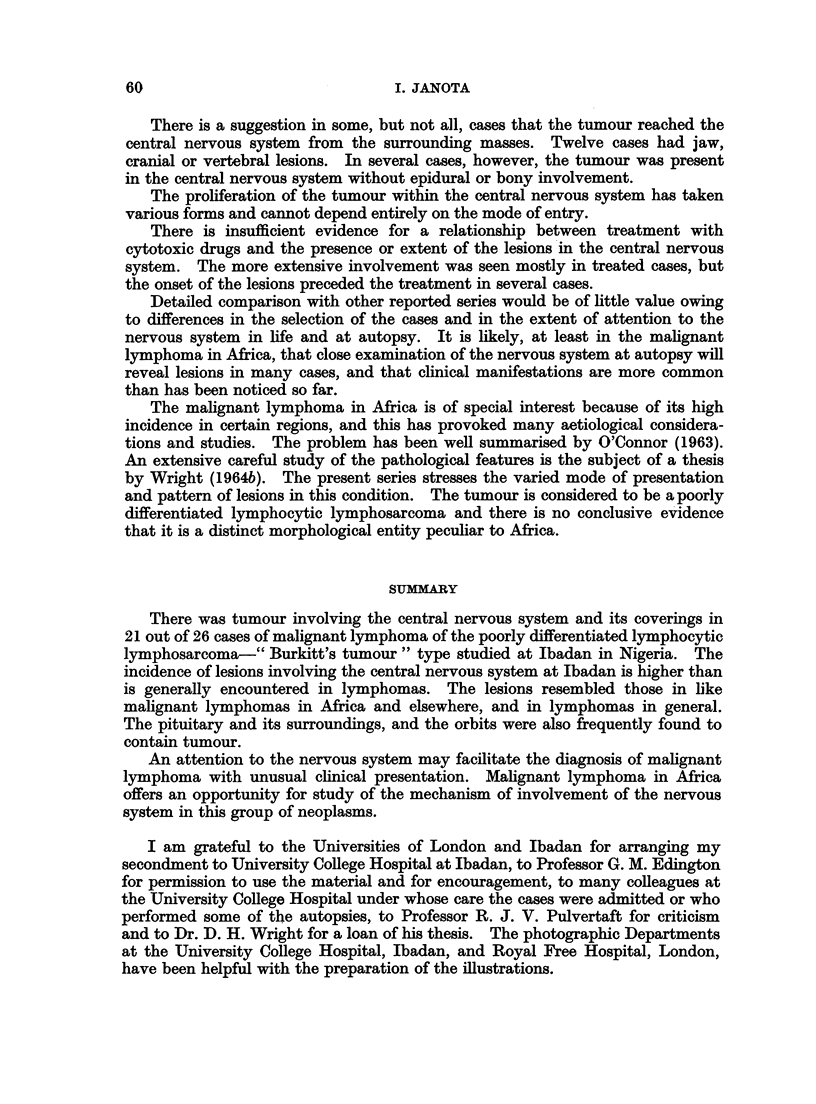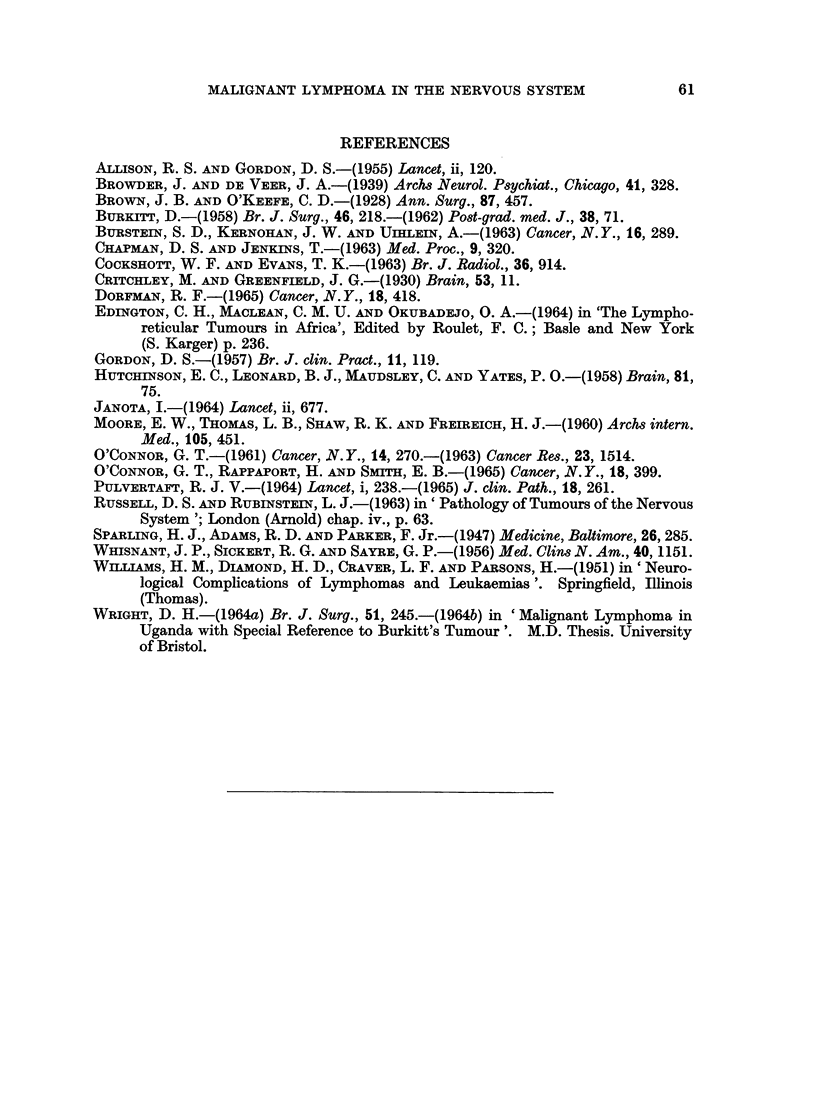# Involvement of the nervous system in malignant lymphoma in Nigeria.

**DOI:** 10.1038/bjc.1966.7

**Published:** 1966-03

**Authors:** I. Janota

## Abstract

**Images:**


### INVOLVEMENT OF THE NERVOUS SYSTEM IN MALIGNANT LYMPHOMA IN NIGERIA

I. JANOTA*

*From the Departments of Pathology, Royal Free Hospital, London, and University College Hospital, Ibadan, Nigeria*

Received for publication November 10, 1965

In recent years a form of malignant lymphoma common in, but not confined to, children has become recognised in Nigeria as elsewhere in equatorial Africa (Burkitt, 1958; Edington, Maclean and Okubadejo, 1964). The commonest presenting features are abdominal or jaw masses and paraplegia. High incidence and the frequent involvement of the jaw are peculiar to malignant lymphoma in Africa. The disease is almost always fatal.

Twenty-one of the 25 cases that came to autopsy in 18 months at University College Hospital at Ibadan in Nigeria showed tumour in the central nervous system and its coverings, a feature unusual in other forms of lymphoma. The impressive frequency of involvement of the central nervous system is the basis of the present study.

The characteristic, but not specific, histological feature of this form of malignant lymphoma is a mass of relatively uniform primitive cells of the lymphoreticular system which may be differentiating towards lymphoblasts, with scattered non-neoplastic histiocytes. It has been classified on the basis of histological examination as malignant lymphoma of the poorly differentiated lymphocytic type (O'Connor, 1961). Poorly differentiated lymphocytic lymphoma or sarcoma, reticulum cell sarcoma of the undifferentiated type, " African " or " childhood " lymphoma, and " Burkitt's " or " Burkitt " tumour are some of the terms employed in the English literature for this tumour. Like cases have been reported in white children in North America, where the tumour is uncommon and rarely involves the jaw (Brown and O'Keefe, 1928; Dorfman, 1965; O'Connor, Rappaport and Smith, 1965). The evidence for establishing " Burkitt's tumour " as a new form of lymphoma on histological grounds is inconclusive. In tissue culture the " Burkitt cells " appear different from those of other malignant lymphomas. They resemble lymphocytes transformed by phytohaemagglutinin, and may represent " a very primitive cell of the lymphocyte series " (Pulvertaft, 1964, 1965).

The findings in 26 cases falling into the poorly differentiated lymphocytic lymphosarcoma—" Burkitt's tumour " category, including one case with tumour in the brain seen earlier at Ibadan (Edington *et al.*, 1964), are here presented.

### *General Comments*

Nineteen of the 26 cases were male and 7 female. Seventeen cases were between 3 and 15, 7 between 16 and 20, and 2 over 21 years old. Jaw involvement was

* Present address: Department of Neuropathology, Institute of Psychiatry, Maudsley Hospital, London, S.E.5.

present in life in 8, and revealed on X-ray examination in further 4 cases. The diagnosis in life was supported in 17 cases by histological examination of biopsy material, with the additional help of marrow biopsy and tissue culture in some. In 3 cases the diagnosis rested on marrow biopsy and tissue culture. In 6 cases, including one where the biopsies were negative, the diagnosis was confirmed only at autopsy. Treatment, which may have influenced the extent of the lesions, was given to 14 cases. It consisted of melphalan, endoxan or nitrogen mustard, given by intraarterial and intravenous injections or orally. Eighteen cases, of whom 9 were treated, survived for less than 6 months from the onset of symptoms. The details of age, sex, duration of disease, and treatment are given in Table I, and the details of diagnostic investigations in life in Table II.

TABLE I.—*Details of age, sex, duration of disease and treatment*

| Case number | Age | Sex | Duration of disease | Duration of treatment |
|---|---|---|---|---|
| 4 | 3 | F. | 4 months | — |
| 12 | 4 | M. | 5 weeks | — |
| 6 | 5 | M. | 2 months | — |
| 11 | 6 | M. | 4 months | 1 day |
| 13 | 6 | F. | 2 months | 6 weeks |
| 1 | 7 | M. | 5 weeks | — |
| 7 | 7 | M. | 3 months | 2 weeks |
| 17 | 7 | M. | 3 months | 1 week |
| 25 | 7 | M. | 3 weeks | 5 months |
| 24 | 8 | F. | 8 months | 5 months |
| 26 | 8 | F. | 9 months | 5 months |
| 18 | 9 | F. | 4 months | 1 day |
| 10 | 10 | M. | 8 months | — |
| 21 | 10 | M. | 10 months | — |
| 22 | 10 | M. | 6 weeks | — |
| 15 | 11 | F. | 5 months | 2 months |
| 20 | 12 | M. | 3 months | 2 weeks |
| 2 | 13 | M. | 15 months | 15 months |
| 19 | 13 | F. | 2 months | — |
| 23 | 15 | M. | 2 months | 2 days |
| 5 | 19 | M. | 3 weeks | — |
| 9 | 19 | M. | 5 months | — |
| 3 | 20 | M. | 2 months | — |
| 16 | 21 | M. | 9 months | 5 weeks |
| 8 | 42 | M. | 7 months | — |
| 14 | 45 | M. | 5 months | 3 weeks |

*Autopsy findings.*—Retroperitoneal tissues and lymph nodes, kidneys, heart and epicardium, and liver showed gross tumour most frequently. In 2 treated cases there was extensive replacement of the adrenals by fibrous tissue without any tumour. Histological examination often revealed tumour in sites where it had not been apparent, notably in the pituitary and its neighbourhood, orbit, pancreas, adrenals and thyroid (Table III).

*Histological appearances.*—The tumour cells at all sites looked unlike mature lymphocytes or reticulum cells. They were larger than lymphocytes, relatively uniform, with a large roughly rounded nucleus, delicate scattered nuclear chromatin, and one or more prominent nucleoli. In some sections there was variation in the size and shape of the nucleus, and notched and lobulated nuclei resembling those in tissue culture (Pulvertaft, 1965) were seen. Close examination revealed vacuoles in the cytoplasm of tumour cells in most cases (Fig. 11). Phagocytic

TABLE II.—*List of investigations in life*

| Case number | Type of investigation: Histology | Marrow biopsy | Tissue culture | Jaw lesions: Gross | X-ray |
|---|---|---|---|---|---|
| 1 | .. | .. | .. | O | .. |
| 2 | + | O | .. | O | ?+ |
| 3 | O | O | .. | O | .. |
| 4 | + | .. | .. | O | ?O |
| 5 | .. | .. | .. | OP | .. |
| 6 | + | O | .. | +H | + |
| 7 | + | O .. | .. | O | + |
| 8 | .. | .. | .. | O | .. |
| 9 | + | .. | .. | O | .. |
| 10 | + | O | .. | O | O |
| 11 | + | O | + | + | + |
| 12 | + | O | + | O | O |
| 13 | + | .. | + | + | .. |
| 14 | + | O | + | O | O |
| 15 | .. | O | + | O | + |
| 16 | + | O | + | O | O |
| 17 | + | O | + | + | + |
| 18 | + | O | .. | O | ?O |
| 19 | .. | .. | .. | + | .. |
| 20 | .. | O | .. | O | + |
| 21 | + | O | .. | O | O |
| 22 | .. | + | + | OHP | + |
| 23 | + | + | + | +H | + |
| 24 | + | + | + | +H | + |
| 25 | .. | .. | + | O | .. |
| 26 | + | .. | O | +H | + |

H indicates occasions when histological examination of the jaw followed the autopsy.
P indicates pain in the jaw.
+ positive finding.
O negative finding.
? indicates doubt.

histiocytes were commonly scattered among tumour cells in the larger masses (Fig. 2 and 3). They contained apparently complete tumour cells and lymphocytes, nuclei, nuclear fragments or brown pigment granules. Some masses, particularly those that shrank on treatment, included a large proportion of histiocytes, as well as reticulin fibres, which were otherwise sparse or absent. The periosteal lesions consisted of tumour elevating the periosteum from the surface of the bone, in places with new bone formation. Even when tumour cells were found in the peripheral blood and in bone marrow biopsy, and the distribution of tumour in the liver resembled that in leukaemia, sections of the bones revealed non-neoplastic haemopoietic tissue alongside tumour unlike in leukaemia (Fig. 4). In the smaller masses tumour cells appeared to have proliferated in the interstitium of the organ, such as thyroid, testis or kidney, with the preservation of apparently normal epithelial structures (Fig. 1).

### CENTRAL NERVOUS SYSTEM AND ITS COVERINGS

The clinical features related to the central nervous system are difficult to assess owing to a varied extent of clinical examination. Paraplegia was present in 4 cases, meningitis complicating bacterial endocarditis in one case, and convulsions or disturbance of consciousness with or without focal signs in 7 cases. Four cases

TABLE III.—*Distribution of lesions in individual cases**

| Case number | Lymph nodes | Retroperitoneal | Kidney | Heart & pericardium | Adrenal | Bones† | Spleen | Liver | Pancreas | Thyroid | Lungs & pleura | Stomach & bowel | Testis | Ovary | Salivary gland |
|---|---|---|---|---|---|---|---|---|---|---|---|---|---|---|---|
| 1 | . . | . . | ● | . . | . . | . . | . . | . . | . . | . . | . . | . . | . . | . . | . . |
| 2 | ● | ● | ● | ● | ● | ● | ● | ● | ● | ● | . . | . . | . . | . . | . . |
| 3 | ● | ● | . . | ● | ● | . . | ● | . . | ● | . . | . . | . . | . . | . . | . . |
| 4 | ● | ● | ● | . . | ● | . . | ● | . . | ● | . . | ● | . . | . . | ● | . . |
| 5 | ● | ● | ● | . . | . . | . . | ● | ● | ● | . . | . . | . . | . . | . . | . . |
| 6 | ● | . . | ● | ● | . . | . . | ● | ● | . . | ● | . . | . . | . . | . . | . . |
| 7 | . . | ● | . . | . . | ● | ● | ● | . . | ● | . . | . . | . . | . . | . . | . . |
| 8 | ● | ● | ● | ● | ● | ● | . . | . . | ● | ● | ● | | | | |
| 9 | ● | . . | . . | . . | ● | . . | . . | . . | ● | . . | . . | ● | . . | . . | . . |
| 10 | ● | . . | . . | ● | . . | ● | . . | ● | . . | ● | ● | ● | ● | . . | . . |
| 11 | . . | . . | ● | ● | . . | . . | . . | . . | . . | . . | ● | . . | . . | . . | . . |
| 12 | ● | ● | . . | . . | . . | . . | . . | . . | . . | . . | . . | ● | . . | . . | . . |
| 13 | . . | . . | ● | ● | ● | . . | . . | ● | ● | ● | . . | . . | . . | . . | . . |
| 14 | ● | ● | ● | ● | ● | ● | ● | ● | . . | ● | ● | ● | . . | . . | ● |
| 15 | ● | . . | ● | ● | ● | ● | . . | . . | . . | . . | ● | . . | . . | ● | . . |
| 16 | ● | . . | . . | . . | . . | . . | . . | . . | . . | . . | . . | . . | . . | . . | . . |
| 17 | ● | ● | . . | ● | . . | . . | . . | . . | . . | ● | . . | ● | ● | . . | . . |
| 18 | ● | ● | . . | ● | ● | . . | ● | . . | . . | . . | . . | ● | . . | ● | . . |
| 19 | ● | ● | ● | . . | . . | . . | ● | ● | . . | . . | . . | . . | . . | . . | . . |
| 20 | ● | . . | ● | . . | . . | ● | . . | ● | . . | ● | . . | . . | ● | . . | ● |
| 21 | . . | . . | . . | . . | . . | . . | . . | . . | . . | . . | . . | . . | . . | . . | . . |
| 22 | ● | ● | ● | ● | ● | ● | ● | ● | ● | ● | . . | . . | ● | . . | ● |
| 23 | ● | . . | ● | ● | ● | ● | . . | ● | . . | ● | . . | . . | ● | . . | . . |
| 24 | . . | . . | ● | ● | . . | ● | ● | . . | . . | . . | . . | . . | . . | ● | . . |
| 25 | . . | ● | ● | . . | ● | ● | . . | . . | ● | . . | ● | ● | . . | . . | . . |
| 26 | . . | ● | ● | ● | . . | . . | . . | ● | . . | . . | ● | . . | . . | ● | . . |
| Total lesions | 18 | 14 | 17 | 15 | 13 | 11 | 11 | 11 | 10 | 10 | 8 | 7 | 5 | 5 | 3 |

* The lesions in and around the central nervous system are presented in Tables IV and V.
† Except jaw and cranium.

had impaired vision, 2 of them in absence of large orbital or jaw tumours. Lumbar puncture was carried out in 9 cases, usually in conjunction with an operation. The cerebrospinal fluid contained tumour cells in Case 26, reported previously (Janota, 1964), and probably in 2 other cases, where the cells have not been correctly identified.

### *Illustrative Cases*

*Case* 6

A 5 year old boy presented 6 days before death with swellings in the left parietal region, behind the left ear, and in the mandible for 2 months. He also had aural discharge, impaired vision and fixed masses in the loins. Biopsy of the parietal mass showed lymphosarcoma. Marrow biopsy was negative. X-rays of jaws showed absence of lamina dura. He was prostrate, vomited, and terminally had fever. The cerebrospinal fluid contained 36 cells per cu.mm. reported as " lymphocytes "—probably tumour cells.

Necropsy showed tumour in the mandible, in many bones of the cranium, the cranial epidural space, the dura, and forming nodules in the inner surface of the dura. The orbits contained no large masses. There was tumour in the submental lymph nodes, epicardium, liver, hepatic veins, inferior vena cava, spleen and kidneys.

Histology confirmed tumour in the mandible, and showed scattered tumour cells posterior to, and in the periphery of, the posterior lobe of the pituitary, in the pituitary stalk, in the soft tissues of the left orbit, in the optic nerve sheath and leptomeninges, and in the orbital fascia. An extensive tumour infiltrate was present in the cerebral leptomeninges and around the cerebral and cerebellar blood vessels. particularly in the floor of the third ventricle. Few clusters of tumour cells were present in the third and fifth cranial nerves.

*Comment.*—This case illustrates diffuse leptomeningeal involvement in association with tumour in the skull in an untreated subject.

*Case* 14

A 45 year old man presented 3 months before death with a history of partial impotence after a fall from a height of 100 feet (30 metres) 6 weeks previously. Three weeks before admission he had pain in the left thigh, and after another week constipation, dribbling of urine, loss of bladder fullness sensation and paraparesis. Examination showed normotonic left and hypotonic right paraparesis, and graded hypoalgaesia from fifth lumbar to fifth sacral segment with normal vibration and position sense. The leg tendon jerks were absent. Myelogram showed a lesion at the level of the second lumbar vertebra. Rectal examination was said to have been negative. At laparotomy soon after admission, an extradural tumour was removed. It was a lymphosarcoma. Tissue culture was positive. X-rays of jaw and bone marrow biopsy were negative. Courses of intravenous and oral endoxan were given. Five weeks before death he had pain in the right subcostal region, 12 days before death he complained of swelling of joints, and 2 days before death the left eye looked prominent. The cerebrospinal fluid contained excess of protein and no excess of cells.

Necropsy showed tumour between the bladder and the rectum extending to the lateral wall of the pelvis, in the left ileo-psoas, in the horseshoe presacral kidney, left adrenal, splenic capsule, abdominal surface of the diaphragm, both pleurae, and the epicardium over the right atrium. There were extensive tumour masses occluding both cavernous sinuses, and in the lumbar spinal canal outside the dura.

Histology also showed tumour in the myocardium, mucosa of the urinary bladder, and thyroid. The pituitary was infarcted with tumour at the periphery and in the stalk. Tumour was present extensively in the leptomeninges, with an ill defined rounded nodule in the depth of the right basal ganglia. There were also tumour cells and necrotic patches in the floor of the fourth ventricle. The spinal cord showed extensive degeneration with loss of anterior horn cells in the lower lumbar region and with dorsal white column demyelination above.

*Comment.*—An adult with paraplegia and an odd nodule of tumour in the basal ganglia.

*Case* 15

An 11 year old girl presented 3 months before death with pain in the left flank, hip, thigh and ear for 2 months, followed by pain in the left eye and a sudden inability to see. She had prominent eyes, bilateral optic atrophy, and, on the one occasion when it was examined, nothing abnormal in the abdomen. X-rays showed small areas of rarification in the sphenoid bones and jaws, and larger areas in the lumbar vertebrae and pubic rami. Sternal bone marrow biopsy was negative. She had a single dose of intraarterial nitrogen mustard 2 months before death. Subsequently she developed flaccid paraplegia. She remained blind.

Necropsy showed no facial swelling. There were tumour masses in the para-aortic lymph nodes, ovaries, around the right and in the left kidney, in both adrenals, in the bodies of the second and third lumbar vertebrae, in the mediastinal and deep jugular lymph nodes and in the surface of the heart. The left occipital lobe of the brain was prominent and felt firm. The spinal cord was adherent to the dura. There were no obvious lesions in the cranium, orbits, spinal canal or pubic rami.

Histology showed tumour also near the main bronchus in the left lung, in the sclera of the left eye, and in the soft orbital tissues on both sides, particularly in the angle between the optic nerves and the eyeballs. There was loss of retinal ganglion cells on both sides. Tumour cells were present at the periphery of the right optic nerve in the optic foramen. Diffuse tumour was present in the adjacent orbital muscles and nerves. Both lobes of the pituitary were infiltrated by tumour which was also present in the adjacent wall of the cavernous sinuses. Clusters of tumour cells were extensively scattered in the pia-arachnoid (Fig. 15) and in the brain, particularly in the left occipital lobe. The cranial nerves, spinal cord, spinal nerve roots, and the dorsal and lumbar epidural space also contained tumour.

*Comment.*—A case with diffuse microscopic involvement of the nervous system, an induration of the brain, and no obvious tumour in the skull bones.

---

### EXPLANATION OF PLATES

Fig. 1.—Tumour in the kidney. A glomerulus and tubules are surrounded by tumour cells. Case 11. H. & E. ×90.

Fig. 2.—Tumour in lymph node. Note histiocyte in the centre. Case 15. H. & E. ×360.

Fig. 3.—Nuclear fragments in the cytoplasm of histiocytes. Epidural spinal tumour. Case 20. H. & E. ×360.

Fig. 4.—Sternum. Dark areas of tumour under the periosteum and in the marrow cavity. The pale areas in the marrow cavity are free of tumour. Case 22. H. & E. ×19.

Fig. 5.—Foci of tumour in the white matter in cerebral hemisphere. The leptomeningeal thickening consists of inflammatory cell infiltrate. Case 17. H. & E. ×7.

Fig. 6.—Tumour in cerebellar leptomeninges and cortex. The white matter is shown along the left border. Case 16. H. & E. ×6.

Fig. 7.—Tumour in both third cranial nerves cut obliquely close to their origin. The mid brain is shown above. Case 16. H. & E. ×6.

Fig. 8.—Longitudinal section of optic nerve showing dark tumour infiltrating in between pale nerve fibres. Case 24. H. & E. ×6·5.

Fig. 9.—Tumour expanding the anterior and posterior nerve roots of the spinal cord. Note pale bundles of nerve fibres surrounded by dark tumour. Case 26. H. & E. ×5.

Fig. 10.—Tumour in temporal lobe. Brain. Case 26. H. & E. ×125.

Fig. 11.—Tumour cells in a perivascular cuff. Note vacuoles in the cytoplasm. Case 17. H. & E. ×880.

Fig. 12.—Extension of tumour from a " cuff " into cerebral white matter. Case 17. H. & E. ×230.

Fig. 13.—Perivascular " cuff " in spinal leptomeninges. Case 15. H. & E. ×230.

Fig. 14.—Biopsy of tumour. Base of brain. Note histiocyte in the centre and scattered dark necrotic cells and cell fragments. Case 21. H. & E. ×230.

Janota.

5

6

7

8

Janota.

Janota.

11

12

13

14

*Case* 16

A 21 year old man presented 6 months before death with pain over sacrum, and in buttocks, back and shoulders for 3 months, and inability to walk and incontinence of urine for 5 weeks. He had left Horner's syndrome, poor grips, flaccid paraplegia, and flexor plantar responses. Tendon jerks and abdominal reflexes were absent. There was hypoalgaesia from the second to fifth dorsal segment, with loss of all sensory modalities below. Abdominal breathing only was present. The anal sphincter tone was lax. Cerebrospinal fluid was yellow and contained much protein. X-rays showed no bony lesion. Myelogram through a cisternal puncture showed a block at the level of fifth cervical vertebra. At laminectomy a 1 cm. thick and 6 cm. long tumour was peeled off the posterior aspect of the dura. Histology showed lymphosarcoma and tissue culture was positive. Subsequently intravenous mustine and endoxan were given. Bone marrow biopsy was negative.

Necropsy 7 days after death showed no obvious tumour at the site of the operation. There were three small tumour nodules in the upper part of the root of the mesentery. The adrenals were enlarged and surrounded and extensively replaced by tough fibrous tissue. The pituitary area looked normal. There was extensive thickening of the basal cerebral and cerebellar leptomeninges. The cranial nerves were very thick, and thick nerve roots packed the space between the dura and the cervical spinal cord.

Histology showed only collagenous fibrous tissue in the cervical epidural area and in the adrenals. The mesenteric tumour was more pleomorphic than the biopsy. A group of tumour cells was present in the anterior lobe of the pituitary. Normal bone marrow was found in the pituitary sella.

There was much tumour in the thickened leptomeninges, nerves (Fig. 7) and nerve roots. In the cerebellum tumour streaked from the leptomeninges into the cortex and beyond (Fig. 6). Tumour was also present in the brain, particularly around blood vessels near the lateral ventricles.

*Comment.*—An adult with extensive involvement of cranial nerves and spinal nerve roots, tumour in the mesentery and curious fibrosis of the adrenals.

*Case* 17

A 7 year old boy presented a month before death with a history of stiff neck and pain in the limbs 2 months earlier, swelling of the left cheek for a month, and recent ankle oedema, abdominal pain and breathlessness. He had rapid pulse and respiratory rate, distended abdomen, enlarged liver, raised jugular venous pressure and systolic cardiac murmur. Later he developed gallop rhythm and a pericardial friction rub. Three weeks before death a hard swelling of the left testis was noticed. Then a swelling appeared in the thyroid isthmus. A biopsy showed a lymphosarcoma and tissue culture " Burkitt cells ". Bone marrow biopsy was negative. X-rays showed patchy absence of lamina dura in the jaws. He was given endoxan. For a few days before death he had left ptosis, left lateral rectus palsy, and a swollen left optic disc with engorged veins and retinal exudates.

Necropsy showed tumour occupying part of the right atrium and involving the adjacent right ventricle, with vegetations on the pulmonary valve. There was also tumour in the right testis, thyroid, mediastinal lymph nodes and left cavernous sinus. Infarcts were present in the left ventricle, lungs and kidneys. There was a diffuse leptomeningeal thickening.

Histology showed bacterial endocarditis, meningitis and septic infarcts, as well as tumour in both testes, thyroid, lymph nodes, serosa of the jejunum and muscle of the appendix, and around the adrenals. There was tumour in the posterior part of the left orbit and in and around the left cavernous sinus extending into the fifth cranial nerve and its ganglion. The pituitary contained no tumour. There was tumour around the blood vessels in the brain, mainly in the white matter (Fig. 5 and 12), in the cerebellum (Fig. 11) and in the cervical spinal cord. The superficial leptomeninges contained, apart from inflammatory infiltrate, cocci and scattered tumour cells.

*Comment.*—This case illustrates unusual presentation with heart failure and a large cardiac mass, bacterial endocarditis and meningitis. The white matter of the brain was infiltrated by tumour.

*Case* 20

A 12 year old boy presented 2 months before death with backache for 10 days, and abdominal pain and ankle swelling and pain for 15 days. He had a papular rash on the face and chest, nodular hepatic mass and an enlarged spleen. He could not stand. Tone, power and tendon jerks in the legs were diminished, and the abdominal reflexes were absent. The various sensory modalities were impaired from the level of the seventh dorsal segment down. Bossing of the left parietal bone, swelling of the left eye, and prominent manubrium sterni were noted. X-rays showed absence of lamina dura in the jaws. Needle biopsy of the sternum, but not of the iliac crest, showed lymphosarcoma. Lumbar puncture yielded fluid with excess of protein and 4 white cells per cu. mm. Intravenous injection of mustine and cyclophosphamide had no effect on the size of the tumour masses and a mandibular swelling became apparent. Seven weeks before death intravenous and oral endoxan was given. Four weeks before death the superficial and palpable tumours all but disappeared. Soon they reappeared and continued to enlarge.

Necropsy showed tumour in the left temporo-parietal region of the skull, in the bones and epidural area of the anterior and middle cranial fossae, mandible, manubrium sterni, in and anterior to the dorsal and upper lumbar vertebrae, in lower spinal epidural space, left orbit, liver, kidneys and some paraaortic and mesenteric lymph nodes.

Histology also showed little tumour in the submandibular salivary gland, testis and thyroid. Tumour was found all around the pituitary. A dorsolateral part of the pituitary was infarcted with a peripheral surviving rim. The cavernous sinus on one side was empty, and on the other side tumour extended around the carotid artery and nearby nerves. The optic chiasma contained much tumour around blood vessels. There was a loss of ganglion cells in the left retina, with small patches of tumour in the optic nerve leptomeninges, extending in between nerve fibres in the optic foramen. There was sparse tumour in the dorso-medial orbital tissues, particularly in between muscle fibres and in small nerves. The brain and spinal cord showed extensive leptomeningeal and perivascular tumour infiltrate, and the cord also showed degeneration due to a lesion in the dorsal region.

*Comment.*—This case illustrates the effect of treatment. The central nervous system, the pituitary, and the surrounding structures were involved by the tumour.

*Case* 21

A 10 year old boy presented $3\frac{1}{2}$ months before death with pain and difficulty in opening the right eye for 7 months, right proptosis and ptosis, fixed pupil, pale optic disc, and, on angiography, a mass in the right fronto-temporal region of the brain. Lumbar puncture yielded cerebrospinal fluid with 10 cells, not identified as tumour cells, per cu.mm. and 90 mg. of protein per 100 ml. Two months before death right ventricular cerebrospinal fluid contained 200 " white " cells per cu.mm. Right osteoplastic craniotomy showed a thinly encapsulated mass displacing the right optic nerve upwards. Biopsy showed a somewhat pleomorphic lymphosarcoma (Fig. 14). After a transient improvement the boy got worse, with fluid accumulating under the bone flap, and developed depressed consciousness, bilateral extensor plantar responses, and Cheyne–Stokes breathing. X-rays showed normal lamina dura. Bone marrow biopsy was negative.

Necropsy was done 24 days after death. There was a large well defined but not sharply demarcated tumour in the right temporal lobe, extending to the pons, and into the frontal lobe, and continuous with necrotic and fibrous roof of the pituitary fossa. There was no tumour anywhere else in the body, apart from a direct extension into the posterior part of the right orbit.

Histology showed a less pleomorphic lymphosarcoma than the biopsy. There was much fibrosis in the cavernous sinuses. The posterior lobe of the pituitary contained some lymphocytes but no tumour cells. There was no tumour in the optic nerves.

*Comment.*—It was impossible to determine the exact site of origin of this isolated intracranial mass, but in absence of bone involvement it is likely that the tumour arose in the brain or in its coverings.

*Case* 22

A 10 year old boy presented 10 days before death with itching of the gums, pain in the legs and loss of weight for a month, inability to walk, swollen abdomen and diarrhoea for 10 days, and sore throat and slurred speech for 5 days. He had *Plasmodium falciparum* in the peripheral blood, was given nivaquin and tetracycline, and was admitted on the next day. He was grossly wasted and weak, with prominent right eye, ill-defined left abdominal mass and palpable right kidney. X-rays showed absence of lamina dura in the jaws and suggested tumour in the shaft and periosteum of the long limb bones and in the iliac crest. Bone marrow from right humerus, iliac crest and tibia showed lymphosarcoma. There were scanty tumour cells in the buffy coat of peripheral blood and tissue culture was positive. The cerebrospinal fluid contained less than 1 white cell per cu.mm and 40 mg. of protein per 100 ml.

Necropsy showed tumour in the pericardium, myocardium, right frontal sinus and orbit, liver, kidneys, spleen, adrenals, in the bones of the skull and cranial epidural space, in the pituitary, in cavernous sinuses, in and over the manubrium sterni, in and anterior to the third lumbar vertebra, and in the shaft and under the periosteum of the left femur. There was a subdural haematoma over the posterior part of the cerebral hemispheres. There were loose tumour masses, not compressing the cord, in the spinal epidural space.

Histology also showed tumour in the pancreas, submandibular salivary gland, thyroid, peritoneum, tongue, rectus abdominis and diaphragm. There were patches of non-leukaemic bone marrow adjacent to tumour in all the bones examined (Fig. 4). In the posterior part of the right orbit there was tumour in the eye muscles and in many of the intramuscular small nerves, but not in the optic nerve. There was also tumour in the angle between the optic nerve and the eyeball. The optic chiasma contained no tumour, but it showed extensive vacuolation. Close examination of the brain and spinal cord showed no tumour, apart from a few cells in the dorsal spinal nerve roots. The tumour in the liver was focal and diffuse.

*Comment.*—Some of the features in this case were suggestive of leukaemia, but the involvement of the bone marrow was patchy. It was odd to see no involvement of the brain or spinal cord with so much tumour all around.

*Case* 24

An 8 year old girl presented 6 months before death with swelling of the mandible and abdomen, impaired vision in the left eye and pain in the arms for 2 months. She had left ptosis. There were masses in both hypochondria. Sternal bone marrow contained tumour, and a biopsy of a skin nodule was suggestive of lymphosarcoma. Tissue culture was positive. X-rays revealed loss of lamina dura. She had intravenous and oral endoxan, and then melphalan. After an initial regression the swellings enlarged in spite of more endoxan. Two weeks before death she had convulsions.

Necropsy showed tumour in the epicardium, kidneys, and ovaries. The adrenals were tough and fibrous. The left optic nerve was thickened.

Histology showed tumour in the heart, lung, left optic (Fig. 8) and many other cranial nerves, spinal nerve roots, leptomeninges, and extensively around the vessels in the brain, cerebellum and spinal cord. The pituitary contained no tumour. A cheek nodule contained only histiocytes.

*Comment.*—As in Case 16, the cranial nerves and spinal nerve roots were extensively involved and the adrenals fibrosed. Both cases have received treatment.

*Case* 26

An 8 year old girl presented 5 months before death with painful swelling of the left side of the face for 4 months followed by bulging of the palate and of the left eye. A mass was palpated in the right lower abdominal quadrant. X-rays showed extensive rarification of the skull, with loss of lamina dura in the maxilla, and osteolytic patches in the ischia. Biopsy of the facial mass showed lymphosarcoma. Tissue culture was not done. She was treated with melphalan and cyclophosphamide. Two weeks before death she had convulsions and after that she was in coma. Two days before death lumbar puncture yielded sterile cerebrospinal fluid with no red blood cells, 50 tumour cells per cu.mm and 300 mg. of protein per 100 ml.

Necropsy showed masses in the ovaries, kidneys, liver and spleen. The bones of the face and of the floor of the cranium were extensively involved by tumour, which had invaded the left temporal lobe of the brain to a depth of 2 cm. There were small opacities throughout the leptomeninges. The third and fifth cranial nerves and the spinal nerve roots were thickened (Fig. 9).

Histology showed tumour in all the sites listed, notably in the temporal (Fig. 10) and frontal lobes, and in leptomeninges, nerves and nerve roots. Small clusters of tumour were present around the cerebral and spinal blood vessels, in the anterior lobe of the pituitary, the orbital muscles and fat, the pericardium and the retroperitoneal fat.

*Comment.*—This case was briefly reported previously (Janota, 1964) together with another one where tumour cells were recognised in the cerebrospinal fluid in life.

### *Findings in the Central Nervous System*

At autopsy there were macroscopical abnormalities related to the central nervous system and its coverings in 15 cases. In a further 6 cases tumour was revealed by histological examination (Tables IV and V).

TABLE IV.—*Distribution of lesions in and around the central nervous system*

| Case number | Site: Jaw and cranium | Orbit | Peripituitary tissues | Pituitary | Cranial dura and epidural area | Brain, leptomeninges and cranial nerves | Spinal dura and epidural area | Spinal cord leptomeninges and nerve roots | Remarks |
|---|---|---|---|---|---|---|---|---|---|
| 1 | O | .. | .. | .. | + | + | .. | .. | Large mass in brain |
| 21 | O | + | + | O | + | + | .. | O | |
| 14 | O | O | + | + | + | + | ± | OL | Ill defined mass in brain |
| 11 | + | + | + | + | + | + | .. | .. | |
| 15 | O | + | + | + | + | + | +V | O | |
| 16 | O | O | O | ± | O | + | + | + | Thickened nerves, nerve roots and leptomeninges |
| 24 | + | + | O | O | + | + | + | + | |
| 17 | + | + | + | O | + | + | .. | ± | |
| 6 | + | + | + | + | + | + | .. | + | Tumour in skull and epidural space |
| 7 | + | .. | ± | O | + | + | .. | O | |
| 20 | + | + | + | + | + | + | +V | + | |
| 22 | + | + | + | + | + | O | +V | ± | |
| 23 | + | + | + | + | + | + | + | + | |
| 26 | + | + | + | + | + | + | + | + | |
| 2 | ?+ | .. | + | + | + | +L | .. | .. | Limited examination, or slight leptomeningeal tumour |
| 4 | ?O | .. | + | O | .. | +L | .. | .. | |
| 8 | O | .. | + | O | .. | ±L | .. | .. | |
| 12 | O | .. | .. | .. | .. | ± | .. | .. | |
| 18 | ?O | + | + | O | O | ± | .. | O | |
| 19 | + | O | + | O | + | + | .. | .. | |
| 25 | O | + | + | O | O | O | O | O | Negative or no examination |
| 5 | O | .. | + | ± | .. | OL | .. | .. | |
| 9 | O | .. | O | O | .. | OL | .. | .. | |
| 10 | O | .. | O | O | .. | OL | .. | .. | |
| 13 | + | .. | .. | .. | + | .. | .. | .. | |
| 3 | O | .. | + | O | .. | .. | .. | .. | |

V—vertebral tumour
L—limited examination
+—positive
±—Slight involvement
O—negative
blank—no examination

TABLE V.—*Incidence of tumour in and around the central nervous system*

| Site | Number of cases | Number of cases examined |
|---|---|---|
| Brain, leptomeninges, cranial nerves, spinal cord, spinal nerve roots | 19 | 24 |
| Cranial dura and epidural area | 11 | 17 |
| Peripituitary tissues and pituitary | 19 | 21 |
| Soft orbital tissues | 10 | 14 |

*Material and methods*

In 17 cases the whole brain was available for histological examination, and in 14 of these the spinal cord or parts of it as well. In 6 cases, where no abnormality was apparent at autopsy, limited amount of brain was preserved. In Case 1 only two blocks of the intracranial tumour were available. In 2 cases the brain was discarded when it appeared normal at autopsy.

The pituitary was generally removed with its surrounding tissues. It was sectioned in 21 and the contents of the orbits in 14 cases.

All sections were stained with haematoxylin and eosin. Selected sections were stained with Van Gieson, phosphotungstic acid haematoxylin and Loyez techniques, and impregnated for reticulin by Gordon and Sweet method. In several cases, frozen sections were impregnated with silver carbonate following modified Penfield's method for glial cells. In each of these tumour from outside the nervous system was used as a control.

*Gross findings*

In 2 cases there were large tumours in the brain involving the basal dura, but apparently not the skull or the jaw. Ill-defined localised induration of the brain was found in 2 other cases. In 3 cases the leptomeninges, and in 2 of these the cranial and spinal nerve roots, were thickened. Masses in the skull extending into the cranial epidural area were encountered in 6 cases. In one of these, Case 26, tumour extended through the basal dura into the brain, and the cranial nerves and spinal nerve roots were also thickened. In the 4 cases of paraplegia there were spinal epidural masses, in 2 of them with other macroscopic abnormalities. In 2 of the cases of paraplegia there was tumour in the vertebral bodies, and in 3 in the retroperitoneal tissues.

*Histological findings*

The tumour was most frequently present in the leptomeninges (Fig. 6) with " cuffs " around superficial leptomeningeal vessels (Fig. 13). " Cuffs " were also common around vessels deep in the white and grey matter, and in several cases the tumour cells appeared to have spread from the " cuffs " into the brain (Fig.12). In 2 cases intense " cuffing " extended over an ill-defined area noticed on naked eye examination, and in Case 17 with associated meningitis, the white matter was selectively involved (Fig. 5).

A well defined patch of tumour with brain elements scattered in it, superficially resembling a microglioma, was found in the basal ganglia in one case.

In the 3 cases with macroscopic masses in the brain, the centre consisted of tumour cells only, but at the periphery there was perivascular " cuffing " and streaking of tumour cells into the surrounding brain. In one of these cases, the cerebral tumour was in continuity with epidural and cranial bone mass. In Case 21 the tumour was present without any obvious lesion outside the central nervous system, thus resembling in presentation a primary reticulum cell sarcoma or microglioma of the brain. There was however no histological support for either diagnosis (Fig. 14), and the pattern of growth was like that in Case 1 with a coexisting renal lesion.

When cranial nerves and spinal nerve roots were involved (Fig. 7, 8 and 9), apparently normal nerve fibres were separated by the tumour.

Tumour was occasionally found in the nerves outside the dura, notably in the small orbital nerves, and in the interstitium of orbital and other muscles.

The epidural cranial and spinal space contained tumour with or without obvious lesions in the related bones or leptomeninges. In the cases with paraplegia, there were no large masses within the dura. Diffuse meningeal (Fig. 6) and nerve root involvement (Fig. 7) in Case 16 was preceded by a single epidural mass responsible for paraplegia and removed at laminectomy. At autopsy tumour was found only in the mesentery, and the adrenals were extensively replaced by fibrous tissue. Diffuse involvement of the body by tumour in Case 25 did not include the brain or leptomeninges.

The peripituitary tissues contained tumour in 18 cases, the pituitary in 10 cases, with an infarction of the anterior lobe in 2, and the orbit in 11 cases.

### DISCUSSION

Paraplegia is a presenting feature in a proportion of cases of the malignant lymphoma in equatorial Africa, and other clinical and histological forms of involvement of the central nervous system have been noticed (Burkitt, 1962 ; Chapman and Jenkins, 1963 ; Cockshott and Evans, 1963 ; Edington *et al.*, 1964 ; O'Connor, 1961 ; and Wright, 1964*a* and 1964*b*). Lesions in the central nervous system were found in North American cases of malignant lymphoma resembling the African variety (Dorfman, 1965). The lesions in both the African and North American cases are like those known to occur in lymphomas in general, and particularly in lymphosarcoma (Allison and Gordon, 1955 ; Browder and de Veer, 1939 ; Burstein, Kernohan and Uihlein, 1963 ; Critchley and Greenfield, 1930 ; Gordon, 1957 ; Hutchinson *et al.*, 1958 ; Moore *et al.*, 1960 ; Russell and Rubinstein, 1963 ; Sparling, Adams and Parker, 1947 ; Whisnant, Sickert and Sayre, 1956 ; and Williams *et al.*, 1951).

The autopsy findings in the group of 26 cases reported here show a higher incidence of lesions related to the central nervous system than most other series of lymphomas. The present series draws attention to the variety and frequency of lesions in and around the nervous system. Localised masses in the brain were reminiscent of primary reticulum cell sarcoma or microglioma. Diffuse cranial nerve involvement and spinal nerve root infiltration by tumour recalled a unique case of giant follicular lymphoma (Russell and Rubinstein, 1963).

The paraplegia in association with retroperitoneal, vertebral and epidural masses is probably due to the combined effect of interference with the blood supply and compression of the spinal cord.

There is a suggestion in some, but not all, cases that the tumour reached the central nervous system from the surrounding masses. Twelve cases had jaw, cranial or vertebral lesions. In several cases, however, the tumour was present in the central nervous system without epidural or bony involvement.

The proliferation of the tumour within the central nervous system has taken various forms and cannot depend entirely on the mode of entry.

There is insufficient evidence for a relationship between treatment with cytotoxic drugs and the presence or extent of the lesions in the central nervous system. The more extensive involvement was seen mostly in treated cases, but the onset of the lesions preceded the treatment in several cases.

Detailed comparison with other reported series would be of little value owing to differences in the selection of the cases and in the extent of attention to the nervous system in life and at autopsy. It is likely, at least in the malignant lymphoma in Africa, that close examination of the nervous system at autopsy will reveal lesions in many cases, and that clinical manifestations are more common than has been noticed so far.

The malignant lymphoma in Africa is of special interest because of its high incidence in certain regions, and this has provoked many aetiological considerations and studies. The problem has been well summarised by O'Connor (1963). An extensive careful study of the pathological features is the subject of a thesis by Wright (1964*b*). The present series stresses the varied mode of presentation and pattern of lesions in this condition. The tumour is considered to be a poorly differentiated lymphocytic lymphosarcoma and there is no conclusive evidence that it is a distinct morphological entity peculiar to Africa.

### SUMMARY

There was tumour involving the central nervous system and its coverings in 21 out of 26 cases of malignant lymphoma of the poorly differentiated lymphocytic lymphosarcoma—" Burkitt's tumour " type studied at Ibadan in Nigeria. The incidence of lesions involving the central nervous system at Ibadan is higher than is generally encountered in lymphomas. The lesions resembled those in like malignant lymphomas in Africa and elsewhere, and in lymphomas in general. The pituitary and its surroundings, and the orbits were also frequently found to contain tumour.

An attention to the nervous system may facilitate the diagnosis of malignant lymphoma with unusual clinical presentation. Malignant lymphoma in Africa offers an opportunity for study of the mechanism of involvement of the nervous system in this group of neoplasms.

I am grateful to the Universities of London and Ibadan for arranging my secondment to University College Hospital at Ibadan, to Professor G. M. Edington for permission to use the material and for encouragement, to many colleagues at the University College Hospital under whose care the cases were admitted or who performed some of the autopsies, to Professor R. J. V. Pulvertaft for criticism and to Dr. D. H. Wright for a loan of his thesis. The photographic Departments at the University College Hospital, Ibadan, and Royal Free Hospital, London, have been helpful with the preparation of the illustrations.

### REFERENCES

ALLISON, R. S. AND GORDON, D. S.—(1955) *Lancet*, ii, 120.

BROWDER, J. AND DE VEER, J. A.—(1939) *Archs Neurol. Psychiat., Chicago*, **41**, 328.

BROWN, J. B. AND O'KEEFE, C. D.—(1928) *Ann. Surg.*, **87**, 457.

BURKITT, D.—(1958) *Br. J. Surg.*, **46**, 218.—(1962) *Post-grad. med. J.*, **38**, 71.

BURSTEIN, S. D., KERNOHAN, J. W. AND UIHLEIN, A.—(1963) *Cancer, N.Y.*, **16**, 289.

CHAPMAN, D. S. AND JENKINS, T.—(1963) *Med. Proc.*, **9**, 320.

COCKSHOTT, W. F. AND EVANS, T. K.—(1963) *Br. J. Radiol.*, **36**, 914.

CRITCHLEY, M. AND GREENFIELD, J. G.—(1930) *Brain*, **53**, 11.

DORFMAN, R. F.—(1965) *Cancer, N.Y.*, **18**, 418.

EDINGTON, C. H., MACLEAN, C. M. U. AND OKUBADEJO, O. A.—(1964) in 'The Lymphoreticular Tumours in Africa', Edited by Roulet, F. C.; Basle and New York (S. Karger) p. 236.

GORDON, D. S.—(1957) *Br. J. clin. Pract.*, **11**, 119.

HUTCHINSON, E. C., LEONARD, B. J., MAUDSLEY, C. AND YATES, P. O.—(1958) *Brain*, **81**, 75.

JANOTA, I.—(1964) *Lancet*, ii, 677.

MOORE, E. W., THOMAS, L. B., SHAW, R. K. AND FREIREICH, H. J.—(1960) *Archs intern. Med.*, **105**, 451.

O'CONNOR, G. T.—(1961) *Cancer, N.Y.*, **14**, 270.—(1963) *Cancer Res.*, **23**, 1514.

O'CONNOR, G. T., RAPPAPORT, H. AND SMITH, E. B.—(1965) *Cancer, N.Y.*, **18**, 399.

PULVERTAFT, R. J. V.—(1964) *Lancet*, i, 238.—(1965) *J. clin. Path.*, **18**, 261.

RUSSELL, D. S. AND RUBINSTEIN, L. J.—(1963) in ' Pathology of Tumours of the Nervous System '; London (Arnold) chap. iv., p. 63.

SPARLING, H. J., ADAMS, R. D. AND PARKER, F. Jr.—(1947) *Medicine, Baltimore*, **26**, 285.

WHISNANT, J. P., SICKERT, R. G. AND SAYRE, G. P.—(1956) *Med. Clins N. Am.*, **40**, 1151.

WILLIAMS, H. M., DIAMOND, H. D., CRAVER, L. F. AND PARSONS, H.—(1951) in ' Neurological Complications of Lymphomas and Leukaemias '. Springfield, Illinois (Thomas).

WRIGHT, D. H.—(1964*a*) *Br. J. Surg.*, **51**, 245.—(1964*b*) in ' Malignant Lymphoma in Uganda with Special Reference to Burkitt's Tumour '. M.D. Thesis. University of Bristol.